# Survey on ureTEric draiNage post uncomplicaTed ureteroscopy (STENT)

**DOI:** 10.1002/bco2.48

**Published:** 2020-10-08

**Authors:** Nikita R. Bhatt, Kenneth MacKenzie, Taimur T. Shah, Kevin Gallagher, Keiran Clement, William A. Cambridge, Meghana Kulkarni, Graeme MacLennan, Rustom P. Manecksha, Oliver J. Wiseman, Samuel Mcclinton, Daron Smith, Veeru Kasivisvanathan

**Affiliations:** ^1^ Department of Urology Norfolk and Norwich University Hospital Norwich UK; ^2^ Department of Urology Sunderland Royal Infirmary Sunderland UK; ^3^ Division of Surgery and Cancer Imperial College London London UK; ^4^ Department of Urology Western General Hospital Edinburgh UK; ^5^ Department of Urology Royal Alexandra Hospital, Urology Paisley UK; ^6^ University of Edinburgh Edinburgh UK; ^7^ Department of Urology Guy's and St Thomas' and King's College London London UK; ^8^ University of Aberdeen The Centre for Healthcare Randomised Trials Aberdeen UK; ^9^ Department of Urology Tallaght University Hospital Dublin Ireland; ^10^ Department of Urology Cambridge University Hospitals NHS Trust Cambridge UK; ^11^ Department of Urology Aberdeen Royal Infirmary Aberdeen UK; ^12^ Department of Urology University College London Hospital NHS Foundation Trust London UK; ^13^ Division of Surgery and Interventional Science University College London; ^14^ British Urological Researchers in Surgical Training (BURST) Collaborative

**Keywords:** randomized controlled trial, ureteric stent, ureteral stent, ureteric stones, urolithiasis

## Abstract

**Objectives:**

To assess the feasibility of conducting a randomised controlled trial (RCT) to assess whether avoiding ureteric drainage is superior to performing ureteric drainage after Uncomplicated Ureteroscopy and/or Flexible Ureterorenoscopy (URS/FURS) treatment of a urinary tract stone in improving patient reported outcome measures (PROMs) and 30‐day unplanned readmission rates. A secondary objective was to understand current practice of urologists regarding ureteric drainage after uncomplicated URS/FURS (UU).

**Material and methods:**

We undertook an online survey of urologists, circulated amongst members of international urological societies and through social media platforms. Uncomplicated URS/FURS was defined as completion of URS/FURS treatment for a urinary tract stone, with the absence of: ureteral trauma, residual fragments requiring further lithotripsy procedures, significant bleeding, perforation, prior urinary tract infection or pregnancy. The ureteric drainage options considered included an indwelling stent, stent on a string or a ureteric catheter. The primary outcome was to determine the proportion of urologists willing to take part in a RCT, randomising patients after UU to a “no ureteric drainage” arm or ureteric drainage arm. Secondary outcomes included determining in their current practice, the proportion of clinicians performing routine ureteric drainage after UU, the reasons for performing ureteric drainage following UU and their preferred optimal duration for ureteric drainage if it is used. The study was reported according to the Checklist for Reporting Results of Internet E‐Surveys (CHERRIES).

**Results:**

Total of 468 respondents from 45 countries took part in the survey, of whom 303 completed the entire survey (65%). The majority agreed that they would be willing to randomise patients (244/303, 81%) in the proposed RCT. Perceived lack of equipoise to randomise was the most common reason for not being willing to participate (59/303, 19%).

92% (308/335) reported that they use ureteric drainage after UU. This was most often due to wanting to prevent possible complications from post‐operative ureteric oedema (77%) or to aid passage of small fragments (43%). Complexity of the case (i.e. impacted stone 90%) and length of the procedure (46%) were the most important intraoperative factors influencing the decision to use ureteric drainage post procedure. If required, the median stated ideal duration of ureteric drainage was 5 days (IQR: 3–7 days) after UU. If having UU personally, 30% would want no stent postoperatively and over half would prefer a stent on a string.

**Conclusion:**

We have highlighted wide variation in practice regarding ureteric drainage after UU. Our results support the feasibility of an RCT evaluating if no ureteric drainage is superior to ureteric drainage in improving PROMs and 30‐day unplanned readmission rates following UU.

## 1 BACKGROUND

Following *ureteroscopy and/or flexible ureterorenoscopy (henceforth referred to as “URS/FURS”) URS/FURS* with or without laser lithotripsy and removal of a urinary tract stone, a ureteric drain in the form of a stent or ureteric catheter can be placed for postoperative drainage. The rationale for this includes the prevention of obstruction of the ureter by a blood clot or a residual stone which requires a further procedure or to allow recovery of a ureter that may have been affected by the procedure. However, as these factors are less of a concern after an uncomplicated URS/FURS (UU), which applies to most ureteroscopic procedures,^1^ it may be possible to avoid the ureteric drainage altogether.

Avoiding a ureteric drain may be advantageous because ureteric stents have a significant negative impact on patients’ quality of life.^2^, ^3^, ^4^ Pain is the most commonly reported side effect, occurring in most patients, however, stent usage has also been shown to cause urinary symptoms including hematuria, inhibit sexual activity and working ability.^2^, ^3^, ^4^ As a consequence, two out of three patients were dissatisfied with the prospect of requiring future ureteric stent insertion.^2^, ^3^, ^4^ Furthermore, in one meta‐analysis of randomized controlled trials (RCT) of ureteral stenting, the use of stents did not improve stone‐free rate, fever, incidence of urinary tract infection, unplanned medical visits, and requirement for analgesia or late postoperative complications.^5^ An analysis assessing the financial impact of stent‐associated morbidity estimated a median loss of earning per patient of approximately GBP367.^3^

However, there is conflicting evidence, supporting the role of stenting after URS/FURS. In two other meta‐analyses, although unstented patients were less likely to experience symptoms of dysuria or pain, the rate of unplanned medical visits and further admission to hospital rehospitalization was significantly higher in the unstented group.^6^, ^7^ There are several reasons for the conflicting results between systematic reviews: lack of consistency in the studies inclusion criteria, their definitions for URS/FURS and definitions of an “uncomplicated” URS/FURS.

The National Institute for Health and Care Excellence (NICE)^8^ and the European Association of Urology (EAU) guidelines^9^ recommend that clinicians should not leave stents after UU. However, there is still a high tendency (63%‐80%) to perform ureteric drainage after UU,^1^, ^10^ which may reflect an uncertainty on the strength of the evidence addressing this practice,^11^ or the desire by the clinician to avoid a potentially significant complication postoperatively, even accepting that this may be associated with some comorbidity for the patient. A Cochrane review identified a need to conduct well designed, sufficiently powered trials to answer this important question.^11^

Stone disease has a significant impact on a patient's quality of life, with adverse symptoms from ureteric drainage after UU often more bothersome than the original stone itself. Following an UU, the impact that ureteric drainage vs no drainage has on a patient's quality of life should be one of the critical outcomes in a RCT evaluating the necessity of ureteric drainage.^12^ There have been validated patient‐reported outcome measures (PROMs) developed for patients with stone disease which could be useful in evaluating this impact.^13^, ^14^ Though in previous studies, unplanned readmissions and complications were commonly evaluated, there has been no RCT comparing ureteric drainage against no ureteric drainage that has studied the validated surgery‐specific PROMs as the primary outcome. The recent Cochrane review alluded to this as one of the short‐comings in the current evidence.^11^ Evaluating 30‐day unplanned readmission rate is also important and although this may be reflected somewhat in the PROMs, we felt that this should be an additional secondary outcome that should be measured in an RCT.

To assess the feasibility of conducting a RCT on this we decided to survey clinicians’ attitudes and opinions regarding ureteric drainage following UU.

## 2 MATERIALS AND METHODS

This was an international online survey of urologists. The *Survey on ureTEric draiNage post uncomplicaTed ureteroscopy (STENT)* (Online Appendix 1) was reported using the Checklist for Reporting Results of Internet E‐Surveys (CHERRIES) (Online Appendix 2).^15^

### 2.1 Design

The survey was obtained using a combination of sampling techniques.^16^ The first was probability‐based using list‐based sampling via email addresses from national urological societies in United Kingdom (UK) (British Association of Urological Surgeons, BAUS), Ireland (Irish Society of Urology, ISU), the British Urological Researchers in Surgical Training (BURST) mailing list, and the Endourological Society mailing list. To increase the response rate, this was combined with a non‐probability sampling technique. This consisted of an unrestricted self‐selected survey method by including a link to the survey on media platforms including Twitter and online news magazines (*Urology News*, the American Urological Association (AUA) residents’ newsletter and the BAUS 2019 conference programme).

### 2.2 IRB (Institutional Review Board) approval and informed consent process

The survey was exempt from requiring ethical approval, though informed consent was obtained from participants in the survey. The survey was anonymous and responses were stored in a password‐protected REDCAP database hosted at University College London (UCL). Responses were only accessible to three authors in the study group designated a priori.

### 2.3 Development and pretesting

The REDCAP survey was piloted prior to final release. The scope, choice of questions, and format were drafted by NB, DS, VK, and edited by the STENT study group as part of the BURST Research Collaborative peer review process involving internal peer review in the collaborative and external peer review by invited experts in the field.^17^ The formatting, sense, reliability, ease of use, and functionality of the REDCAP survey were tested in several rounds by the STENT study group.

### 2.4 Recruitment process, description of the sample having access to the questionnaire, and survey administration

The survey was first advertised in June 2019 and was open for 3 months from 20/06/19 to 16/09/19. Advertisement was to the sampling frames described above. Urologists and trainee urologists routinely performing URS/FURS were permitted to take part in the survey. Their experience was ascertained objectively as part of the survey.

### 2.5 Preventing multiple entries from the same individual

Instructions to permit only single completion of the survey were given in advance with contact details of the study team in the case of any technical difficulties or questions in order to prevent multiple entries. We collected information on the place of work, timing of survey completion, and whether the respondent was a trainee or a consultant which allowed us to find duplicate records.

### 2.6 Survey content

The participants of the survey were presented with a clinical scenario and asked to comment on their practice in the last month:

*The typical clinical scenario is a patient who has had a definitive routine URS/FURS with or without fragmentation using laser for ureteric or renal stones. Following the procedure there is an opportunity to either place a ureteric stent or other form of ureteric drainage (eg, ureteric catheter) or to decide not to place any postoperative ureteric drainage. Definitive URS/FURS means the final URS/FURS for stone disease (ie, the stone treatment is complete, such that no further stone treatment is anticipated). Uncomplicated means (as based on EAU guidelines) the absence of:*

- *ureteral trauma*,
- *residual fragments*,
- *bleeding*,
- *perforation*,
- *UTIs or*
- *pregnancy*.

The survey had a combination of closed and open‐ended questions containing free text to allow respondents to explain or elaborate their answers (Online Appendix 1). The themes explored included self‐reported frequency of stenting, reasons for stenting and duration of stenting after URS/FURS, and seeking opinion regarding equipoise to participate in a RCT comparing ureteric drainage to no drainage after UU.

### 2.7 Outcomes

#### 2.7.1 Primary outcome

1. The proportion of urologists willing to randomize patients after UU to an arm with no ureteric drainage or to an arm with ureteric drainage

#### 2.7.2 Secondary outcomes

1. The reported frequency of stenting after UU in their current practice
2. Categorized reasons for leaving a stent after UU
3. The proportion of urologists using methods of ureteric drainage (including stent, stent on string, or ureteric catheter) of ureteric drainage after UU in their current practice
4. Urologists’ preferred stated optimum duration of stenting after UU
5. Estimated current typical duration of ureteric drainage in UU in “real life” clinical practice
6. Description of any difference between urologists’ clinical practice, and what they would recommend for themselves or their family.

### 2.8 Analysis

The overall response rate was determined based on the number of fully completed questionnaires. The data reported in partially complete questionnaires were used, we determined the attrition rate by page, screen and question. The questionnaires that were terminated early were analyzed and the questions that were answered were included in the analysis. The data were assessed for normality and descriptive statistics were performed. The qualitative data were analyzed using thematic analysis and the Braun and Clarke method^18^ was used to generate codes that were further analyzed for frequency of occurrence to determine the most common themes in the open‐ended answers.

## 3 RESULTS

### 3.1 Response rates

The email list sampling frame by email consisted of 4671 clinicians comprising 2500 members of The Endourological society, 1337 members of BAUS (British Association of Urological Surgeons), 737 urologists on the BURST mailing list, and 97 members of ISU (Irish Society of Urology). The number of potential respondents will have been less than the total sampling frame due to multiple affiliations to these societies. In addition, the total number of impressions generated from tweets on the survey were 35 549 with 861 engagements. Overall, 468 respondents took part in the survey, of whom 303 (65%) completed all of the questions. Sixty‐nine percent of the respondents (321/468) were consultants, and 31% (147/468) were trainees; of those who completed all the questions 72% (217/303) and 28% (85/303) were consultants and trainees, respectively.

### 3.2 Current practice

The median number of URS/FURS performed per month was 11 (IQR 2‐12) and a quarter performed more than 16 URS/FURS per month (Supplemental material).

**FIGURE 1:**
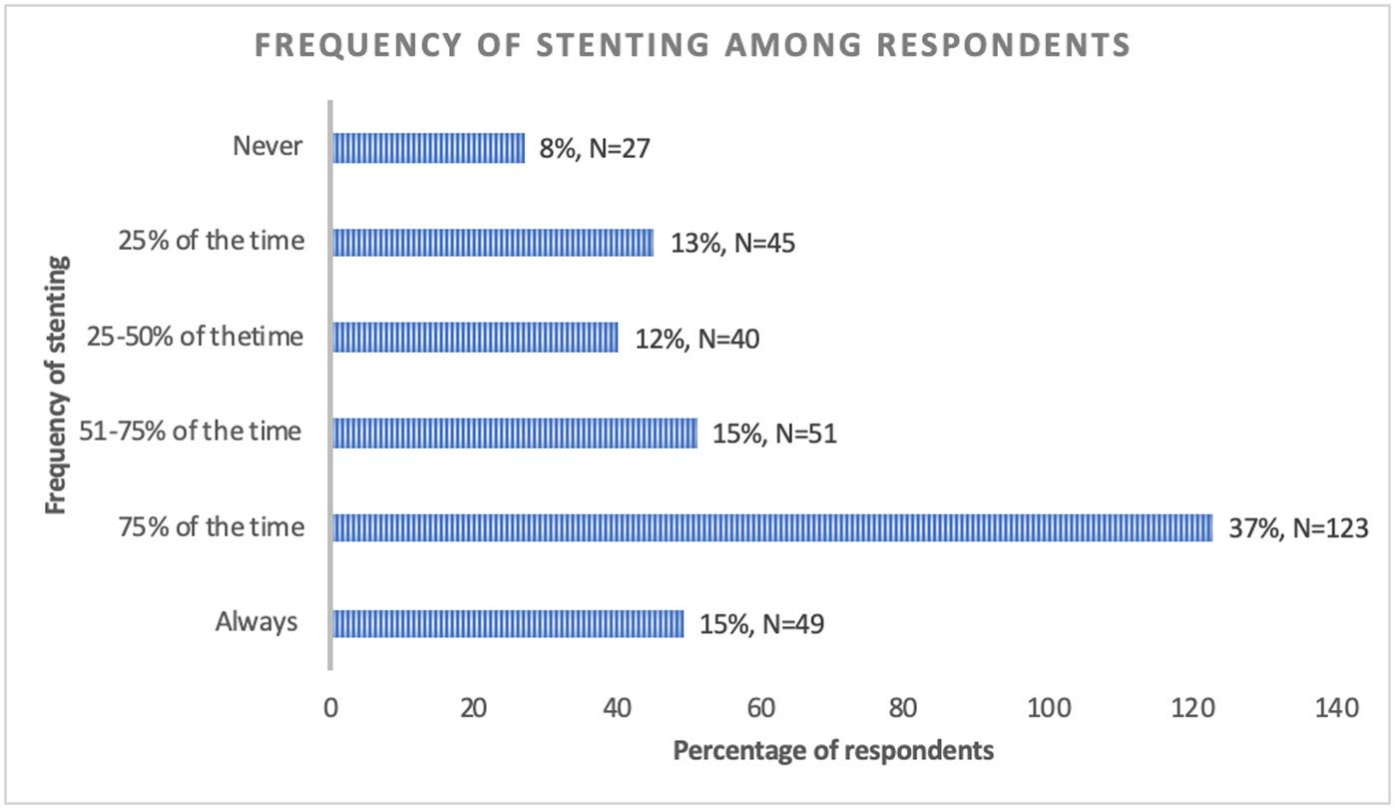
Frequency of inserting a stent after uncomplicated URS/FURS among respondents

Ninety‐two percent (308/335) of urologists reported having performed some form of postoperative ureteric drainage following UU in the past month. Nearly two‐thirds of respondents would more likely than not leave a stent after an UU, whereas only 8% (27/335) would never leave a stent after UU (Figure 1).

**FIGURE 2:**
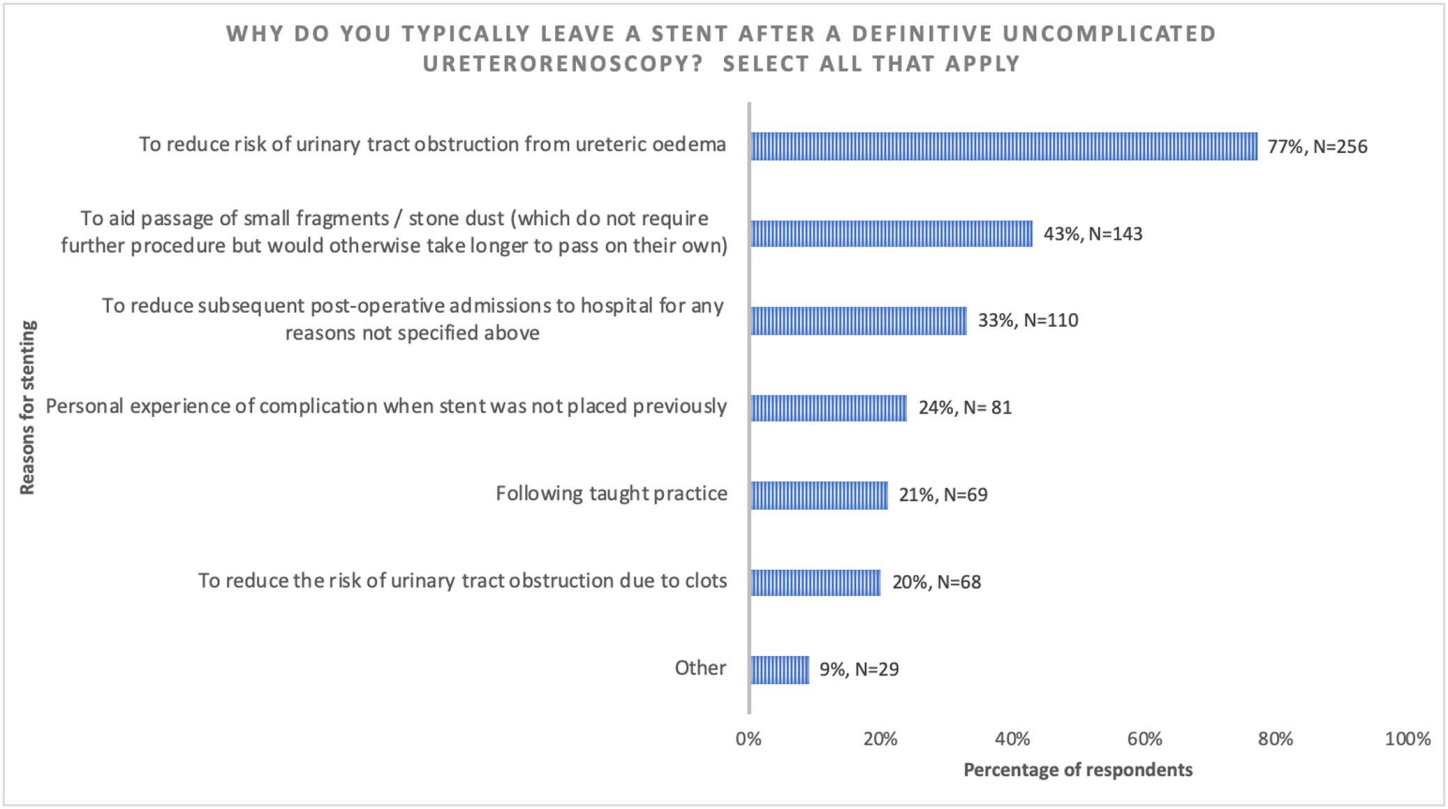
Reasons for leaving a stent after uncomplicated URS/FURS

The most common reason for leaving a stent was the perceived risk of ureteric obstruction from edema (77%, 256/333) with 43% (143/333) leaving a stent to “aid the passage of small fragments” (Figure 2). Of note, only 1% (3/333) of respondents suggested that they left a stent due to defensive practice for medicolegal concerns.

**FIGURE 3:**
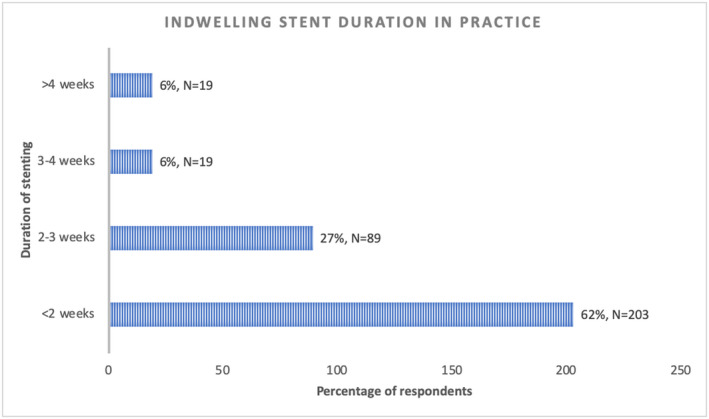
Indwelling stent duration in clinical practice after uncomplicated URS/FURS

In those leaving stents after UU, the median preferred optimal duration was 5 days (IQR 3‐7) while in their actual clinical practice, respondents specified that stents are typically left in for a median of 7 days (IQR 5‐14) (Figure 3). Logistics or resource issues were not a major barrier to timely stent removals in the majority of respondents' centers, with 76% (251/332) stating that this occurred less than a quarter of the time or never. When there was a resource issue, the most common was the lack of capacity to perform an earlier flexible cystoscopy to remove the stent (55/333, 17%).

**FIGURE 4:**
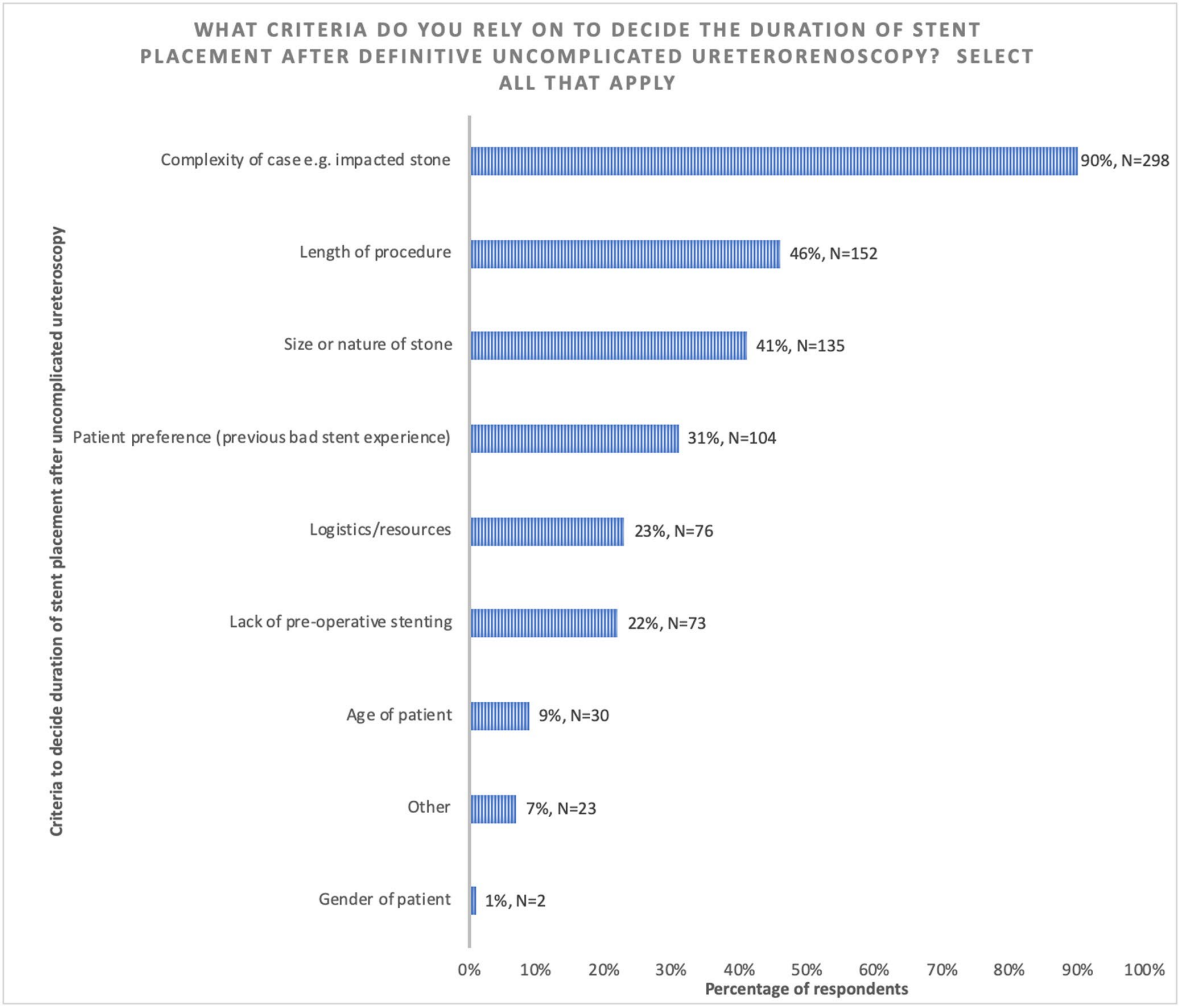
Criteria to decide duration of stent placement after uncomplicated URS/FURS

In those patients whom respondents decide to place a stent following UU, the most common criterion that influenced stent dwell time was the complexity of the case, for example, an impacted stone (298/332, 90%) followed by the duration of the procedure (152/332, 46%) and the size and nature of the stone (135/332, 41%) (Figure 4).

**FIGURE 5:**
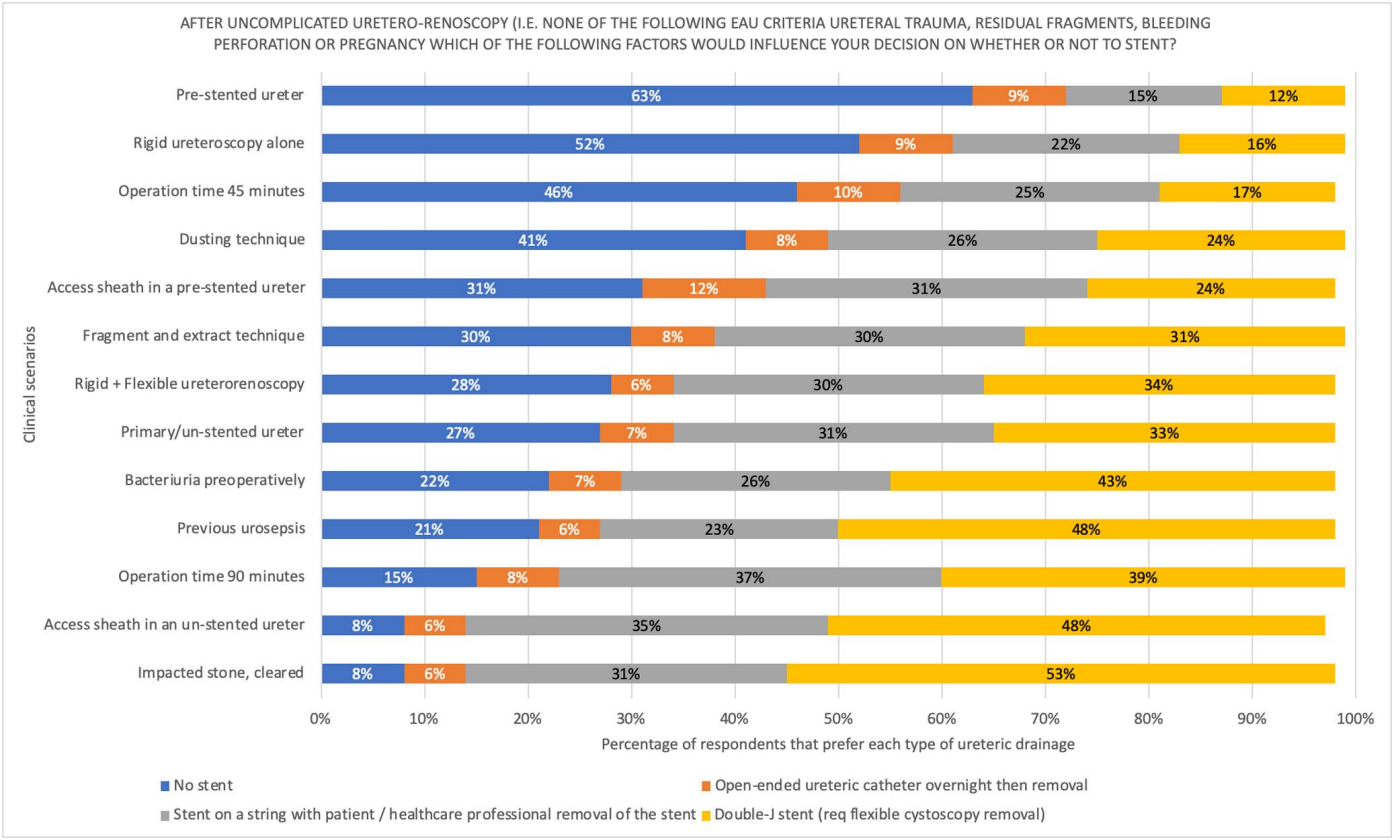
Decision on ureteric drainage after uncomplicated URS/FURS in depending on clinical scenario. Bars represent the proportion of respondents who would use that form of ureteric drainage for each scenario, the coloured bars represent different forms of ureteric drainage (labelled in key)

Figure 5depicts the complexities involved in decision making for ureteric drainage depending on the preoperative and perioperative clinical scenario and demonstrates the type of drainage option preferred, if any, following UU under various preoperative and intraoperative circumstances. Options included ureteric catheter drainage overnight, ureteric stent with the string left on and removal by the patient or by a health‐care professional in the following days‐weeks, or ureteric stenting with flexible cystoscopic removal in the following days‐weeks.

Overall, some form of ureteric drainage was preferred over no ureteric drainage in all scenarios except for two: a pre‐stented ureter and following rigid ureteroscopy alone without ureterorenoscopy. Respondents felt most comfortable not leaving any ureteric drainage in the following scenarios: a pre‐stented ureter (63%), when performing rigid ureteroscopy without ureterorenoscopy (52%), when the total operative time was no more than 45 minutes (46%), and when using a dusting only technique with laser lithotripsy (41%). Respondents felt least comfortable not leaving any ureteric drainage after treating an impacted stone (8%), after using an access sheath in an unstented ureter (8%), and when the operation time reached 90 minutes (15%).

In terms of the types of ureteric drainage options, the least preferred in all circumstances was a ureteric catheter left overnight (range 6%‐12% across various indications). The more complex the clinical scenario, the more likely a respondent would prefer a stent with flexible cystoscopy removal over leaving a stent on a string with removal by the patient or health‐care professional. For the majority of scenarios, a stent with flexible cystoscopy removal was preferred but a stent on a string removed by the patient or health‐care professional was more commonly preferred to flexible cystoscopic when the operation time was no more than 45 minutes (percent difference in favor of stent on a string, 8%), after the use of an access sheath in a pre‐stented ureter (percent difference in favor of stent on a string, 7%) or when rigid ureteroscopy was done alone without URS/FURS (percent difference in favor of stent on a string, 6%).

**FIGURE 6:**
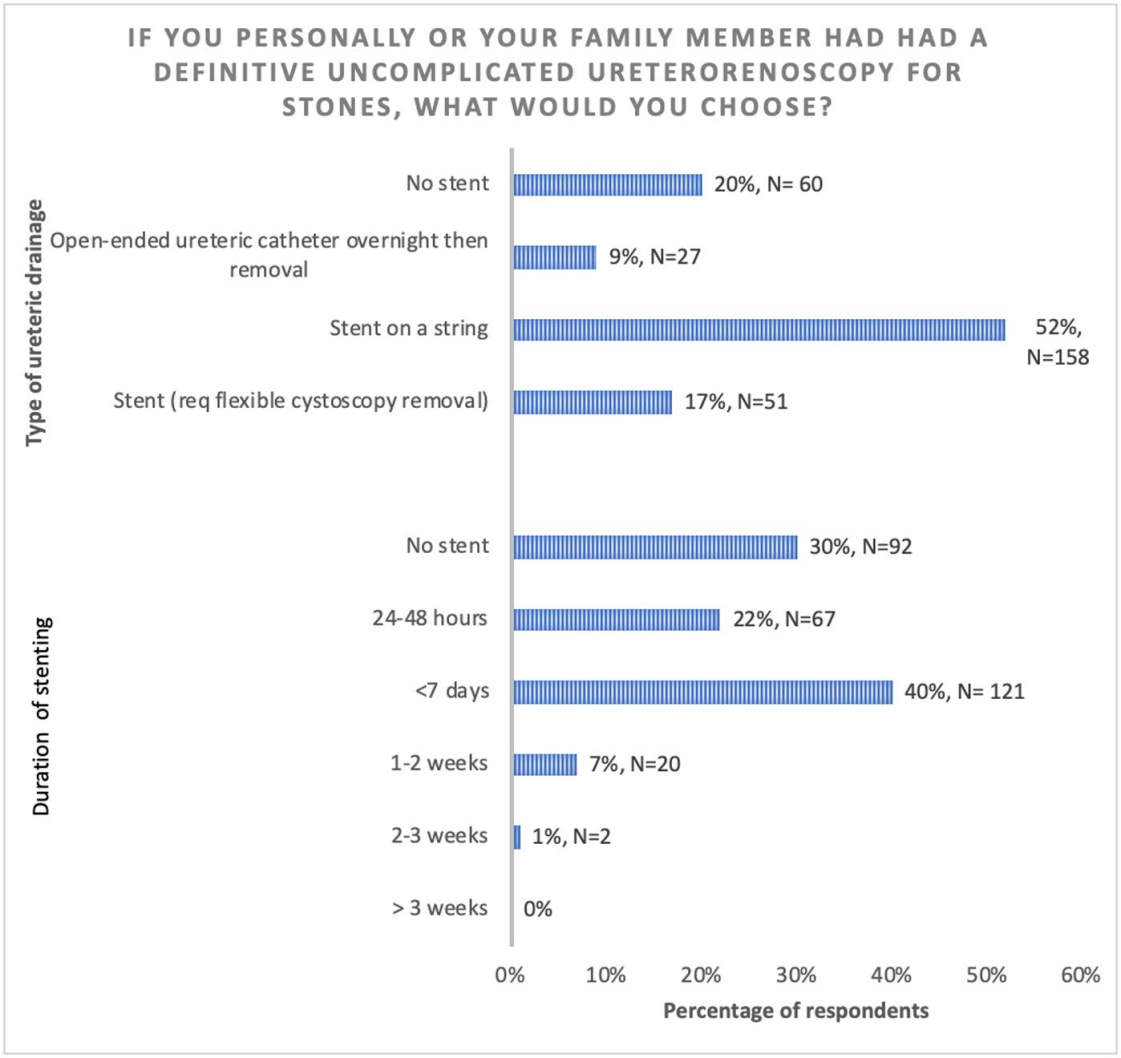
Decision on ureteric drainage after uncomplicated URS/FURS in personal situation

Despite the responses summarized above, when asked to choose a ureteric drainage option if they themselves or their family member underwent an UU, 30% of the respondents would not want any form of ureteric drainage at all. In terms of the type of ureteric drainage option they would chose, the majority (52%) preferred a stent on a string with removal by themselves or a health‐care professional compared to a stent requiring flexible cystoscopy removal (17%) or ureteric catheter (9%) (Figure 6).

### 3.3 Participation in RCT

**FIGURE 7:**
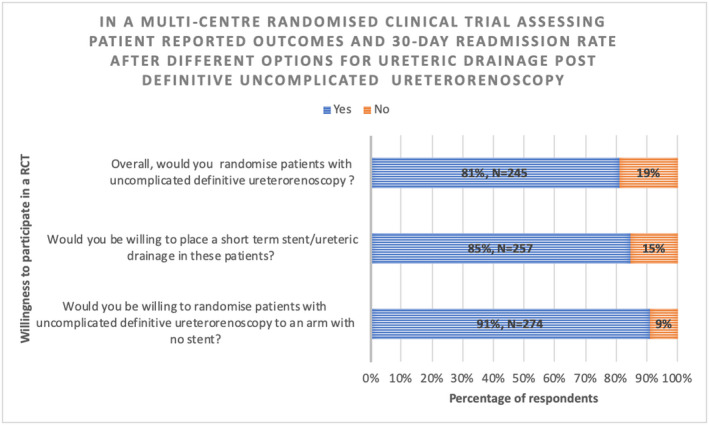
Equipoise to randomize patients after uncomplicated URS/FURS

Eighty‐one percent (244/303) of respondents agreed that they would be willing to randomize patients to an RCT assessing whether no ureteric drainage is superior to ureteric drainage with respect to patient reported outcomes and 30‐day readmission rates (Figure 7). The most common reasons for being unwilling to randomize patients was a perceived lack of equipoise between drainage vs no drainage. Other notable reasons included are detailed in Table 1. When considering all the applicable forms of ureteric drainage that would be reasonable to allow in the RCT, 65% (197/303) thought a stent with flexible cystoscopy removal would be reasonable, 64% (193/303) thought a stent on a string with health‐care professional or patient removal was reasonable, and 27% (81/303) thought a ureteric catheter overnight would be reasonable.

**TABLE 1:** Reasons for not being able to participate in an RCT comparing ureteric drainage vs no drainage after uncomplicated URS/FURS

Reasons for being unwilling to participate in RCT	Frequency
Perceived lack of equipoise	67% (35/54)
Following taught practice	15% (8/54)
Previous bad experience when stents were not inserted	7% (4/54)
Difficulty in getting ethics approval	6% (3/54)
Freedom to make decisions on a case‐by‐case basis	5% (2/54)

## 4 DISCUSSION

The principal finding of this study was establishing sufficient equipoise among clinicians and willingness to randomize patients to a future RCT to assess whether no ureteric drainage after UU is superior to ureteric drainage with respect to PROMs and 30‐day unplanned readmission rates. These findings support the feasibility to conduct such an RCT. We also gained unique perspectives on clinicians’ current practice following UU including the specific criteria that would lead to a lower threshold for postoperative ureteric drainage as opposed to no ureteric drainage, and their ideal choice of ureteric drainage method.

The vast majority of respondents (92%) used some form of ureteric drainage in their routine clinical practice even when URS/FURS is uncomplicated. Clinicians reported that they would use ureteric drainage primarily due to concerns of potential complications from postoperative ureteric edema or to aid passage of small stone fragments. Stents may however impede the passage of stone fragments rather than aid their passage, as evidenced in studies on stenting prior to shockwave lithotripsy.^19^ Despite the high tendency toward using ureteric drainage, the majority of clinicians were still willing to randomize patients in an RCT between no ureteric drainage and ureteric drainage. This highlights that clinicians feel that this is an important research question that could significantly change their practice. It also suggests that they would feel their patients could be safely included in an ethically approved RCT to explore this formally.

Our results show that an RCT comparing stent ureteric drainage with flexible cystoscopic removal of the stent or an arm of stent on a string with health‐care professional or patient removal to no ureteric drainage at all would be feasible. However only a small proportion of urologists considered ureteric drainage catheter left overnight and removed the next day as a suitable option for a large range of clinical scenarios, possibly as it converts an otherwise day‐case URS/FURS into a procedure requiring an overnight stay.

PROMs in urolithiasis patients have garnered interest as a decrease in the general quality of life in stone formers has been reported in the last decade.^13^ Patients with recurrent urolithiasis report more bodily pain, depression, and lower health scores compared to the average population with increased anxiety and stress even between events.^13^ This is compounded by a stone event which may lead to ureteric stenting with its documented negative effect on patients’ quality of life.^2^, ^3^, ^4^ Hence including PROMs in outcome measures while investigating urolithiasis patients is vital.

Previous surveys on ureteric drainage practice after UU have demonstrated a similar high rate of stenting. A prospective multicenter audit of 8 UK centers on 249 patients undergoing URS showed that 74% of patients had some form of ureteric drainage (68% stents and 6% ureteric catheter).^10^ The most common reason to insert a stent was to “protect and dilate the ureter due to concerns about oedema and stone fragments.” Another UK audit by the Endourology section of BAUS on 143 rigid ureteroscopies reported 65% patients were stented and 10% patients had ureteric catheters.^20^ Our results are consistent with surveys of American urologists with 63%‐80% reporting preferring to leave a stent half to all of the time.^1^, ^21^

There is no consensus between international guidelines such as the EAU, AUA, and NICE guidelines on the definition of “uncomplicated” URS/FURS and indications for ureteric stenting. Our survey identified some key factors beyond those that guidelines typically consider, to be considered an uncomplicated ureteroscopic procedure, such as a pre‐stented ureter, performing rigid ureteroscopy without ureterorenoscopy, an operative time of less than 45 minutes, and using a dusting only technique with laser lithotripsy.

There is also no consensus on the optimal stent dwell time or the most appropriate method for stent removal, if one is inserted, following UU. Respondents in our survey felt that the ideal duration of ureteric drainage, when required, was 5 days however in the respondents’ practice, issues related to logistics and resources commonly led to small delays. Animal studies have shown ureteric edema and upper tract obstruction on imaging persist for at least 96 hours after ureteric dilation, though there are no equivalent human studies.^22^ A Japanese study found that stent duration shorter than 14 days was associated with decreased adverse events and lower antibiotic use.^23^ A study that looked at stent duration of 3 vs 7 days showed worse outcomes with 3 days of stenting with an increase in postoperative events and increase in rate of flank pain.^24^

Studies have previously reported that a ureteric catheter for up to 24 hours post URS/FURS may be a reasonable compromise between stenting vs not stenting after UU^25^ though in our study a minority of clinicians chose this as a drainage option. Leaving a stent on a string may have a lower overall stent dwell time with 10% risk of stent dislodgement.^26^ Longer stent dwell duration is a risk factor for stent encrustation and sepsis.^27^ Stent encrustation occurs in 26.8% patients with stent duration less than 6 weeks and increases to 75.9% at more than 12 weeks.^28^ Considering the adverse effect stents can have on patients’ quality of life, it would seem preferable to leave stents in for the shortest duration necessary. However, although it is clear that longer stent dwell times (>4 weeks) are not beneficial and can even be harmful, it is not established whether shorter stent dwell time is effective and can reduce patient morbidity.

Of note, there was disparity in the respondents’ clinical practice and their personal preference for stenting after uncomplicated URS/FURS for themselves. If having such a procedure, 30% would want no stent postoperatively compared to 8% who apply this routinely in their practice. The most commonly preferred option was a stent on a string for less than 7 days. Though clinicians would stent the majority of their patients, the fact that they would not prefer the same treatment for themselves perhaps suggests that many of the stents inserted following UU could be avoided and gives further support for an RCT to investigate this question. Future work should include patient and public member, clinician, and methodologist involvement in the design and development of a pilot RCT comparing whether no ureteric drainage is superior to ureteric drainage with respect to PROMs and 30‐day unplanned readmission rates.

The insights gained from this work on clinicians’ views on the complexity of a URS/FURS procedure for urinary tract stones requiring ureteric drainage may help to redefine criteria for what an uncomplicated URS/FURS may be in contemporary practice. These criteria could be explored and defined in a formal consensus meeting.

There are limitations of this work. Due to the different methods of survey distribution including the use of social media platforms, it was not possible to calculate the exact response rate. However, the utilization of social media platforms is becoming more common in contemporary survey practice,^29^ thus methodology to evaluate surveys distributed in this manner should adapt to accommodate this development in technological advances. One advantage of social media advertisement is to widen the participation and remove barriers to taking part in surveys. Although this is the case, this does mean that those urologists not on social media may be less likely to take part, though our advertising via email and newsletters would have more likely engaged some of these individuals. There is potential selection bias as the respondents are all engaged members of the urology community whose views may differ from those less engaged in surveys or clinical trial work. That said, the aim of this survey was to obtain information from a sample of urologists who are more likely to engage with trials in this disease area and therefore those that did reply are likely to be representative of those that may take part in a future RCT.

## 5 CONCLUSION

This work supports the feasibility of an RCT assessing whether no ureteric drainage after UU is superior to ureteric drainage with respect to PROMs and 30‐day unplanned readmission rates. This work also highlights that urologists' decision to place a ureteric stent is multifactorial and involves components which are not currently included in international recommendations.

## CONFLICT OF INTEREST

The authors of this manuscript have no conflicts of interest to declare.

## Supporting information

Supplementary Material
