# Supplementary material for: Survey on ureTEric draiNage post uncomplicaTed ureteroscopy (STENT)
Source: BJUI Compass. 2020 Oct 8;2(2):115–25. doi: 10.1002/bco2.48 (PMC8988693; doi:10.1002/bco2.48)
Supplement: Supplementary file 1 — Supplementary Material [file BCO2-2-115-s001.docx]

**Supplementary material**

**Appendix 1: Survey on Redcap**


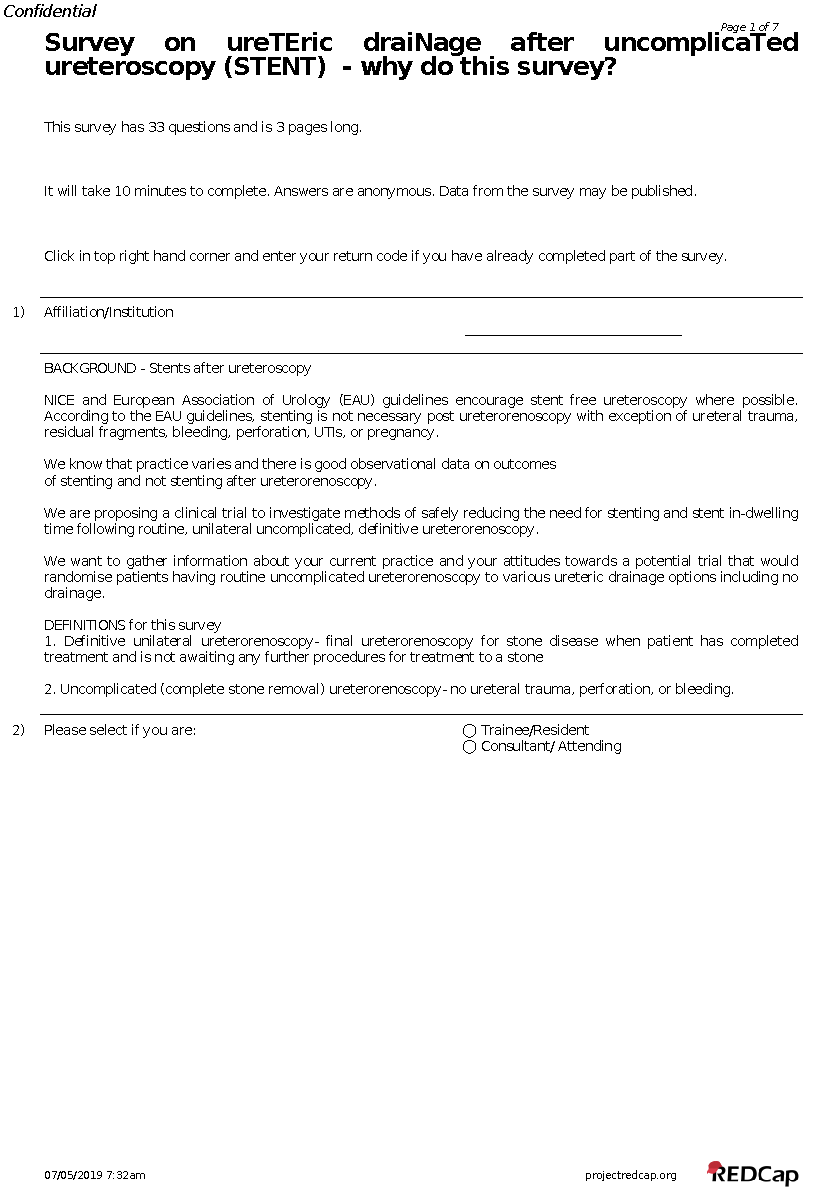


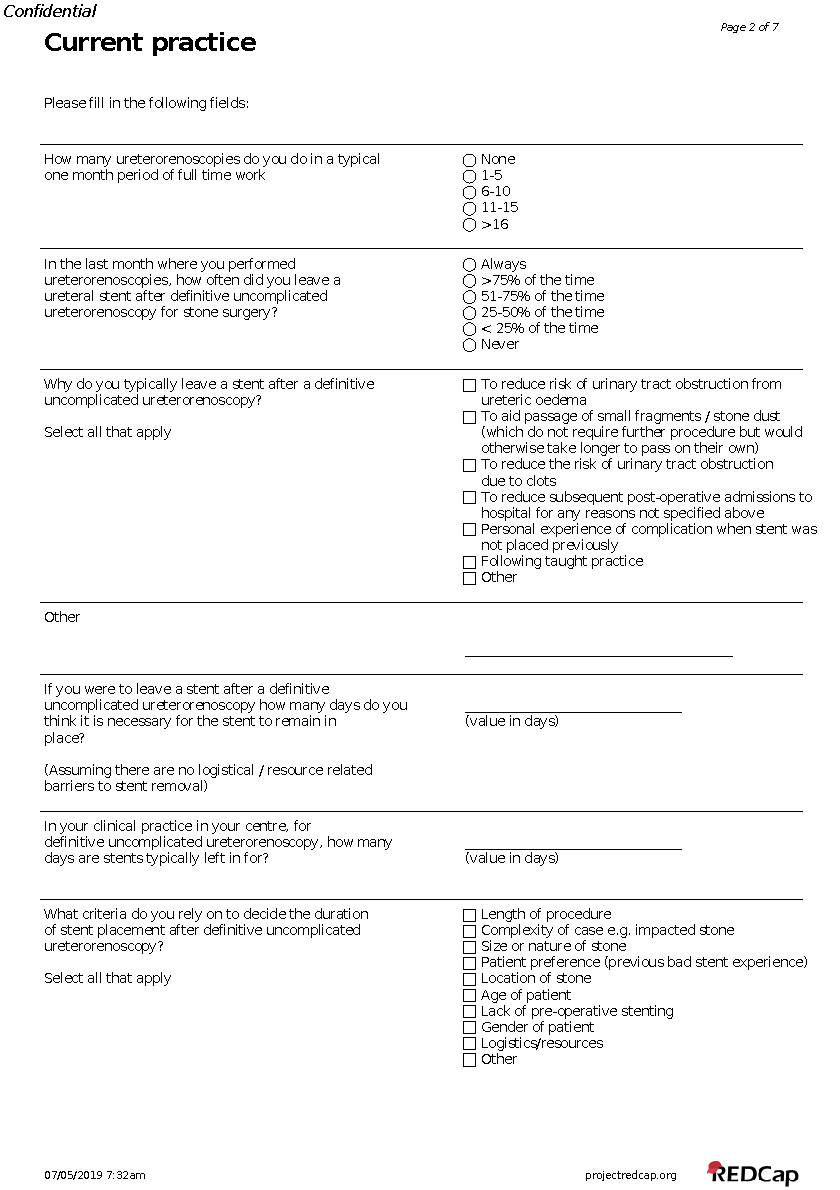

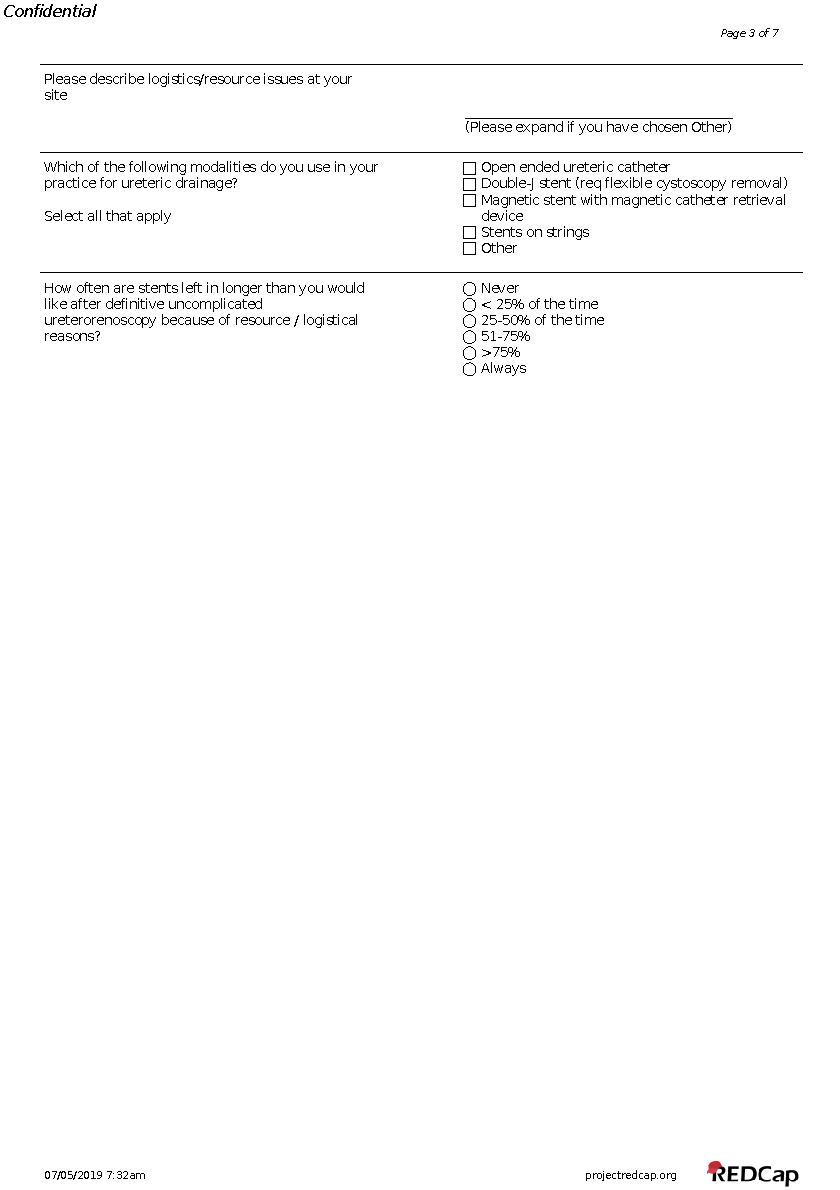

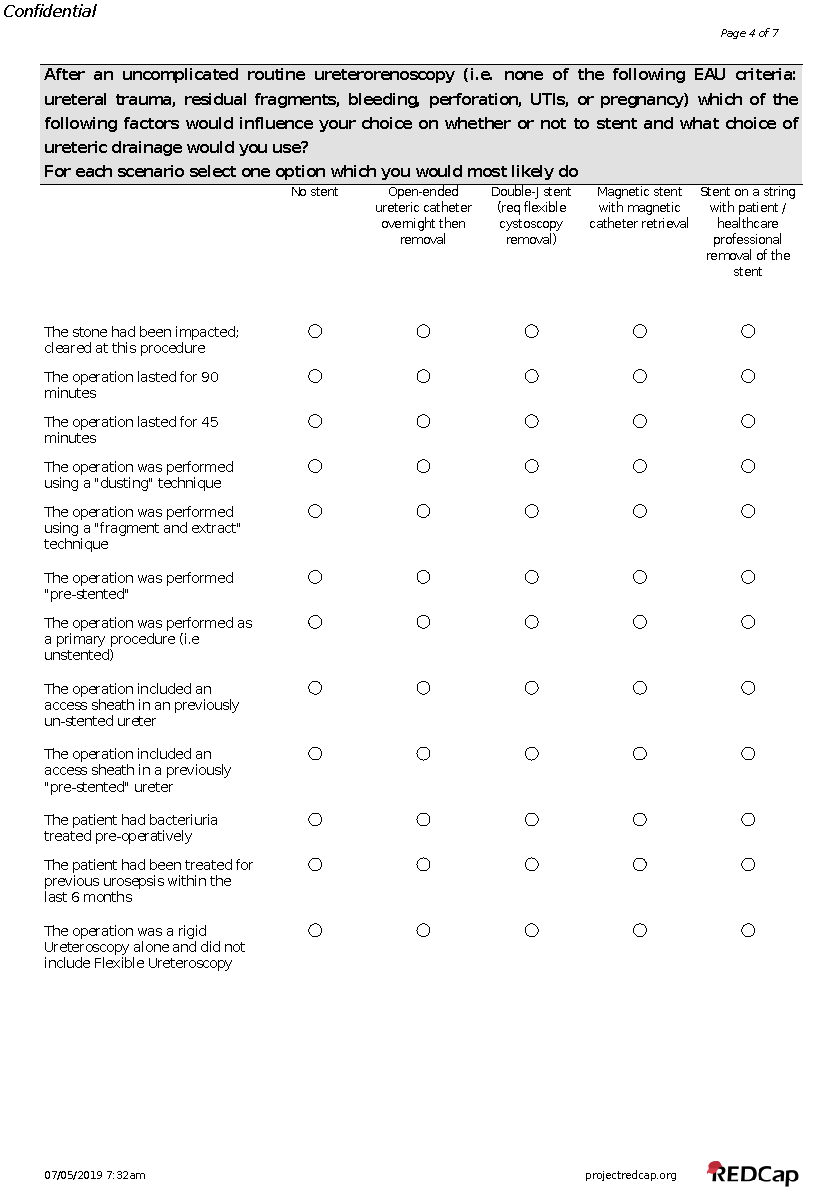

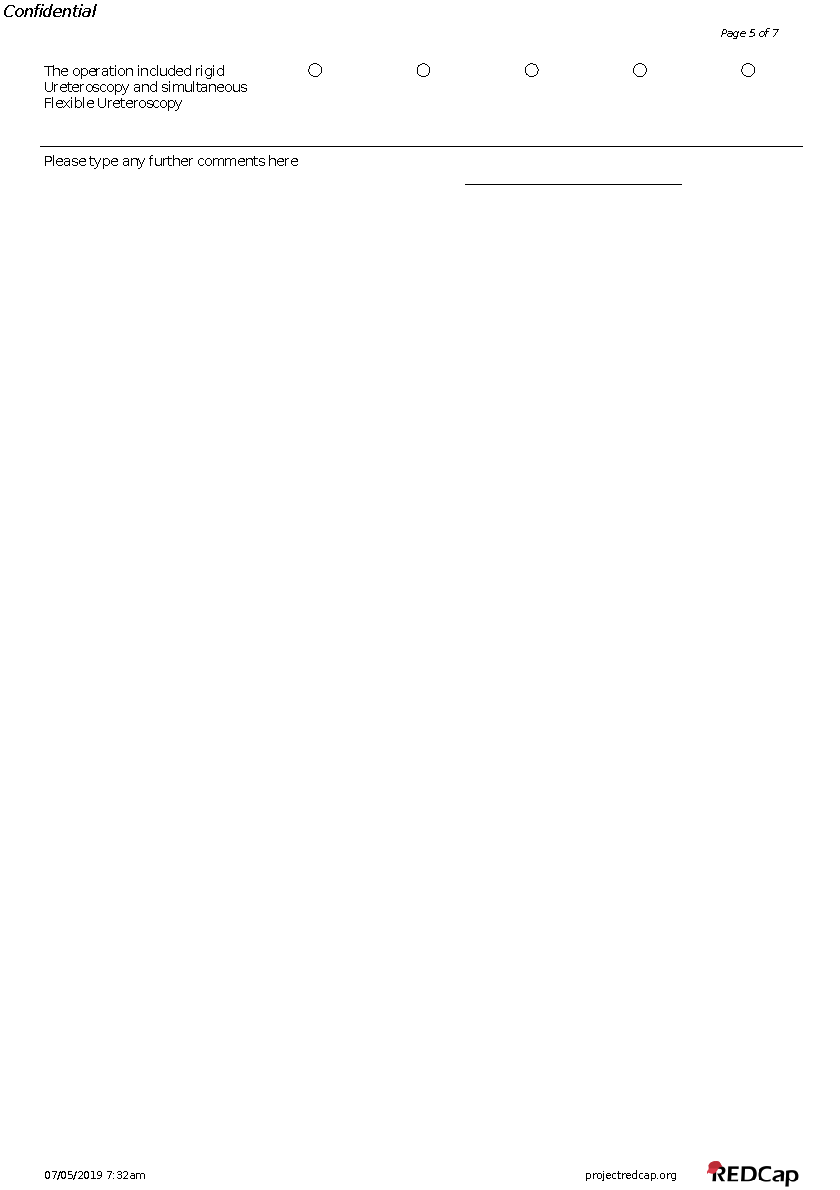

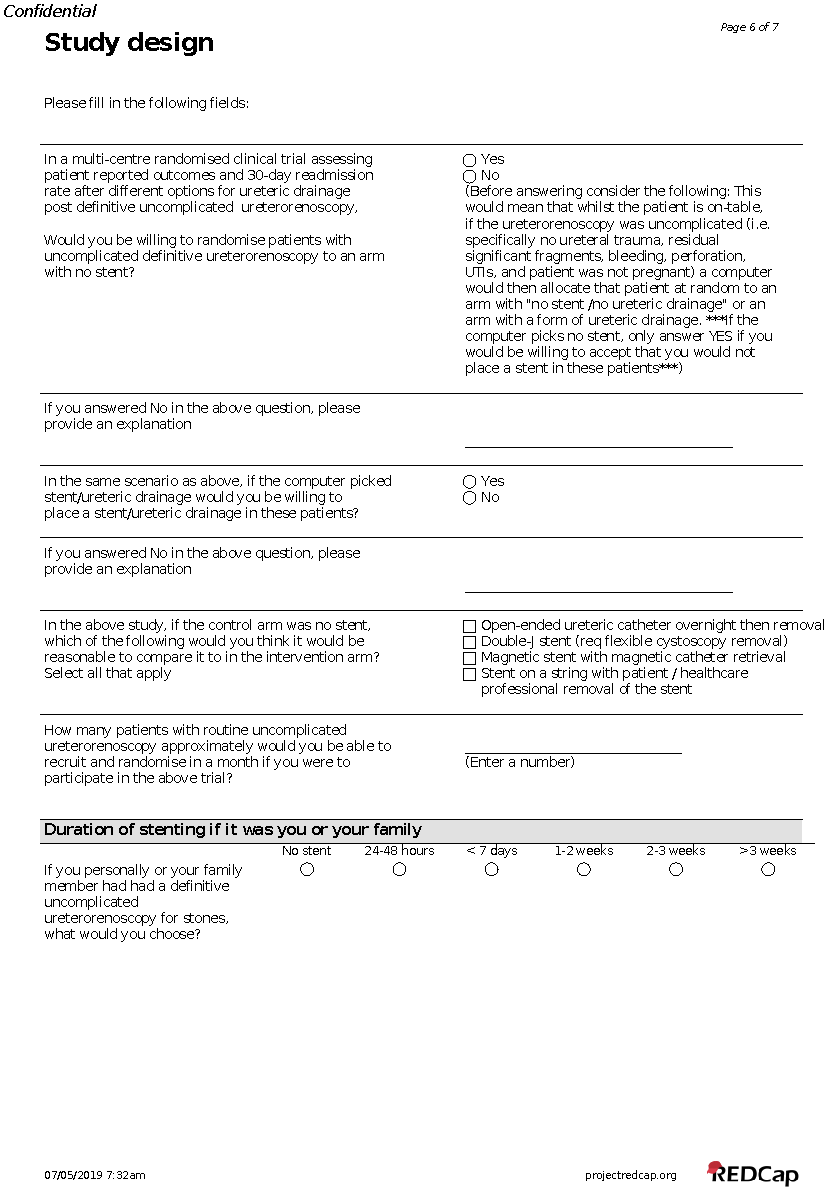

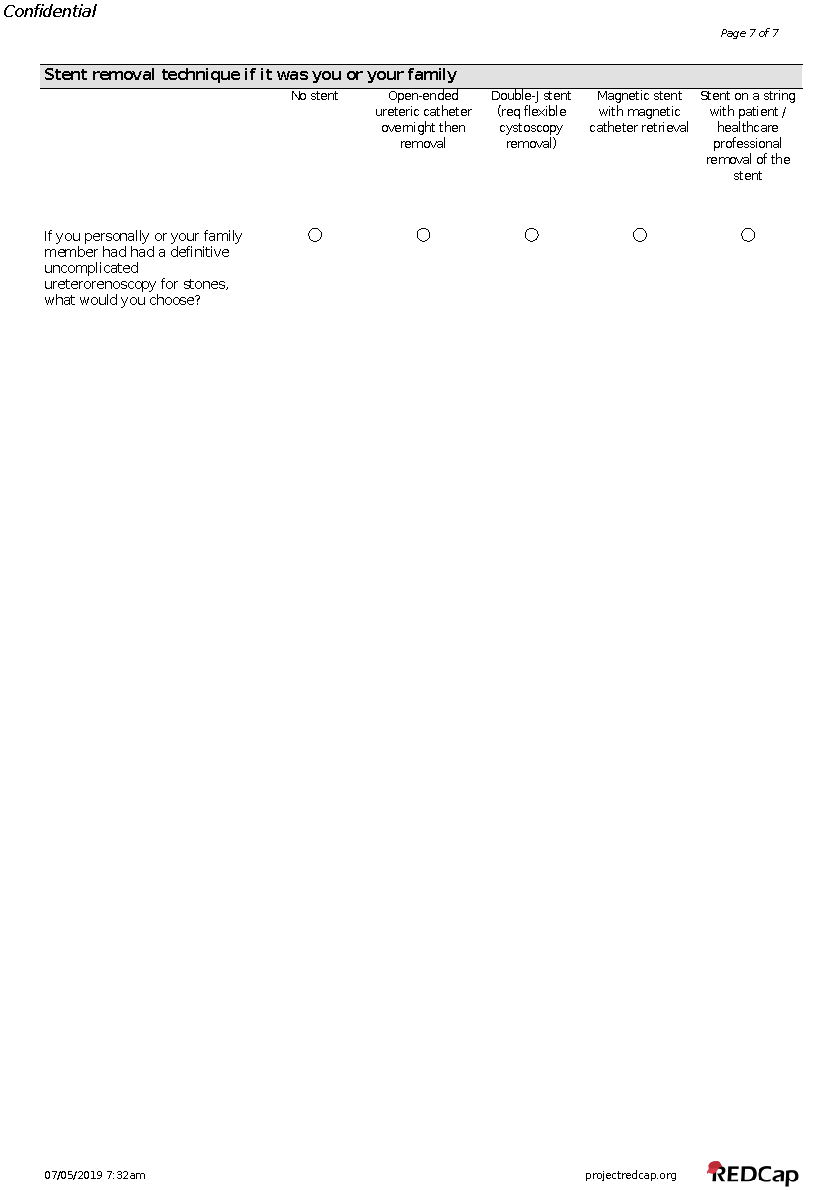


**Appendix 2: CHERRIES checklist for reporting survey**

|  | Checklist for Reporting Results of Internet E-Surveys (CHERRIES) | |
| --- | --- | --- |
| ***Item Category*** | *Checklist Item* | *Explanation* |
| **Design** |  |  |
|  | Describe survey design | The sample was obtained using a combination of sampling techniques. The first was probability-based using list-based sampling via email addresses from various urological societies, this was combined with a non-probability sampling technique i.e. unrestricted self-selected survey method by posting a link to the survey on various platforms like Twitter, online news magazines |
| **IRB (Institutional Review Board) approval and informed consent process** |  |  |
|  | IRB approval | Was not required as this was an online survey circulated globally |
|  | Informed consent | Informed consent was obtained in the beginning by attaching a cover note describing the survey, its purpose, desired outcomes and approximate time taken to complete it |
|  | Data protection | The responses were stored in a password protected computer, the secure redcap database is hosted at University College London (UCL) and was only accessible by three authors who had access to the results through their password protected accounts. |
| **Development and pre-testing** |  |  |
|  | Development and testing | The REDCAP survey was piloted prior to final release. The scope, choice of questions and format was drafted by NB, DS, VK and edited by other authors as part of the BURST Research Collaborative peer review process involving internal peer review in the collaborative and external peer review by invited experts in the field |
| **Recruitment process and description of the sample having access to the questionnaire** |  |  |
|  | Open survey versus closed survey | This was open survey |
|  | Contact mode | Initial contact with the potential participants was made on the internet |
|  | Advertising the survey | The survey was extensively advertised on Twitter and following that during the BAUS meeting in the “BURST session” and in the final highlights session. The link to the survey was also available on the conference program app and the BURST webpage. |
| **Survey administration** |  |  |
|  | Web/E-mail | This was an e-survey with a link posted on the BURST website in addition to emails to potential participants. REDCAP was used to capture all responses |
|  | Context | The BURST website is the online webpage detailing the activities of the BURST group and would be visited by urologists or researchers interested in the activities of BURST |
|  | Mandatory/voluntary | The survey was voluntary and filled out by people who were interested |
|  | Incentives | No monetary incentives were offered, the survey results would be shared with all participants |
|  | Time/Date | Survey remained open for nearly 12 weeks |
|  | Randomization of items or questionnaires | To prevent biases items can be randomized or alternated. |
|  | Adaptive questioning | Adaptive questioning was applied using the branching logic in REDCAP |
|  | Number of Items | The first page was a cover letter, the second page contained 12 items based on current practice and the third page contained 7 items based on future trial design |
|  | Number of screens (pages) | The survey was designed over three pages or screens online, |
|  | Completeness check | The survey was built to have mandatory responses to all questions but, respondents were allowed to save their work and return to complete this at a later stage which may be a factor leading to incomplete responses |
|  | Review step | Respondents were able to review and change their answers using a Back button |
| **Response rates** |  |  |
|  | Unique site visitor | Unique identifier feature was not used for this survey in redcap, the only identifiers available were the place of work and whether the respondent was a trainee or a consultant which helped to determine duplicate records to some extent. |
|  | View rate (Ratio of unique survey visitors/unique site visitors) | We have no way of counting this due to the varied ways of advertising the survey through social media and newsletters |
|  | Participation rate (Ratio of unique visitors who agreed to participate/unique first survey page visitors) | We have no way of counting this due to the varied ways of advertising the survey through social media and newsletters |
|  | Completion rate (Ratio of users who finished the survey/users who agreed to participate) | A total of 468 respondents completed their demographic data, of the 468 respondents that started the survey, 303 completed all questions |
| **Preventing multiple entries from the same individual** |  |  |
|  | Cookies used | We did not use any cookies |
|  | IP check | We did not use IP address of the client computer to identify potential duplicate entries from the same user |
|  | Log file analysis | We did not use any log file analysis. |
|  | Registration | There was no username but the user could record their entry number and click the link again to fill their survey using their original entry number |
| **Analysis** |  |  |
|  | Handling of incomplete questionnaires | Questionnaires which terminated early (where, for example, users did not go through all questionnaire pages) were analyzed |
|  | Questionnaires submitted with an atypical timestamp | We did not use any timestamps to analyse questions |
|  | Statistical correction | We did not use propensity scores or weighting of items |

**Supplemental material**


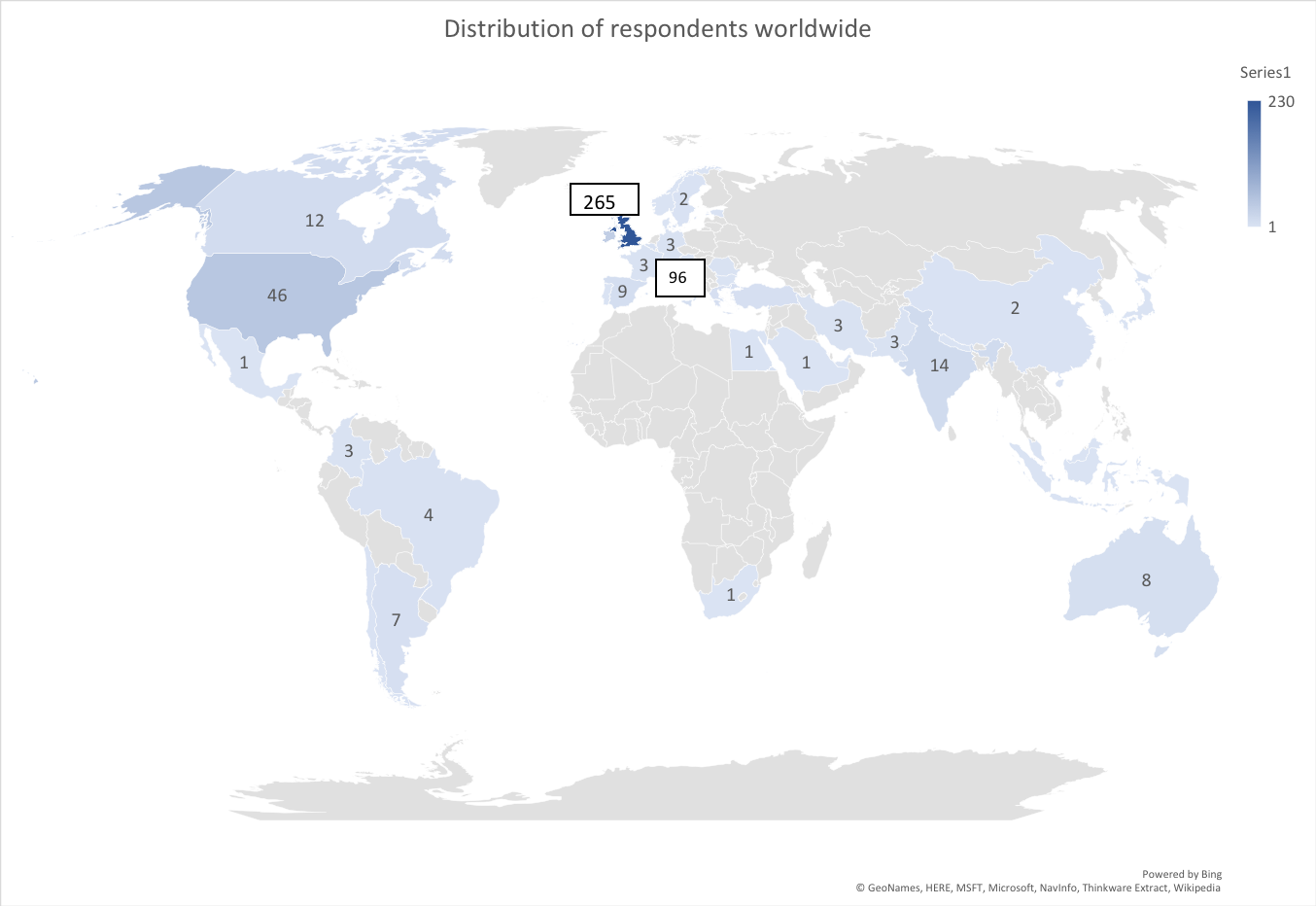


*Supplementary Figure A: Distribution of survey respondents worldwide*


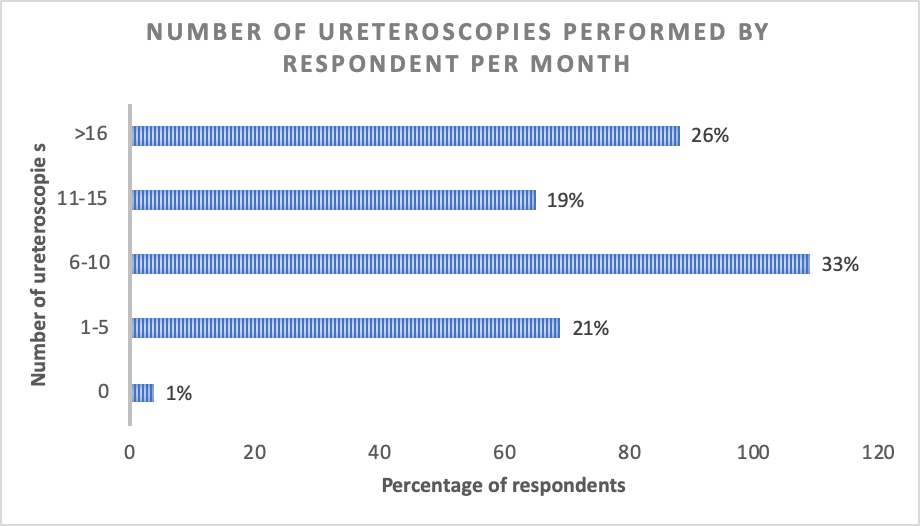


*Supplementary Figure B: Ureteroscopies performed by respondent in a typical month of practice*


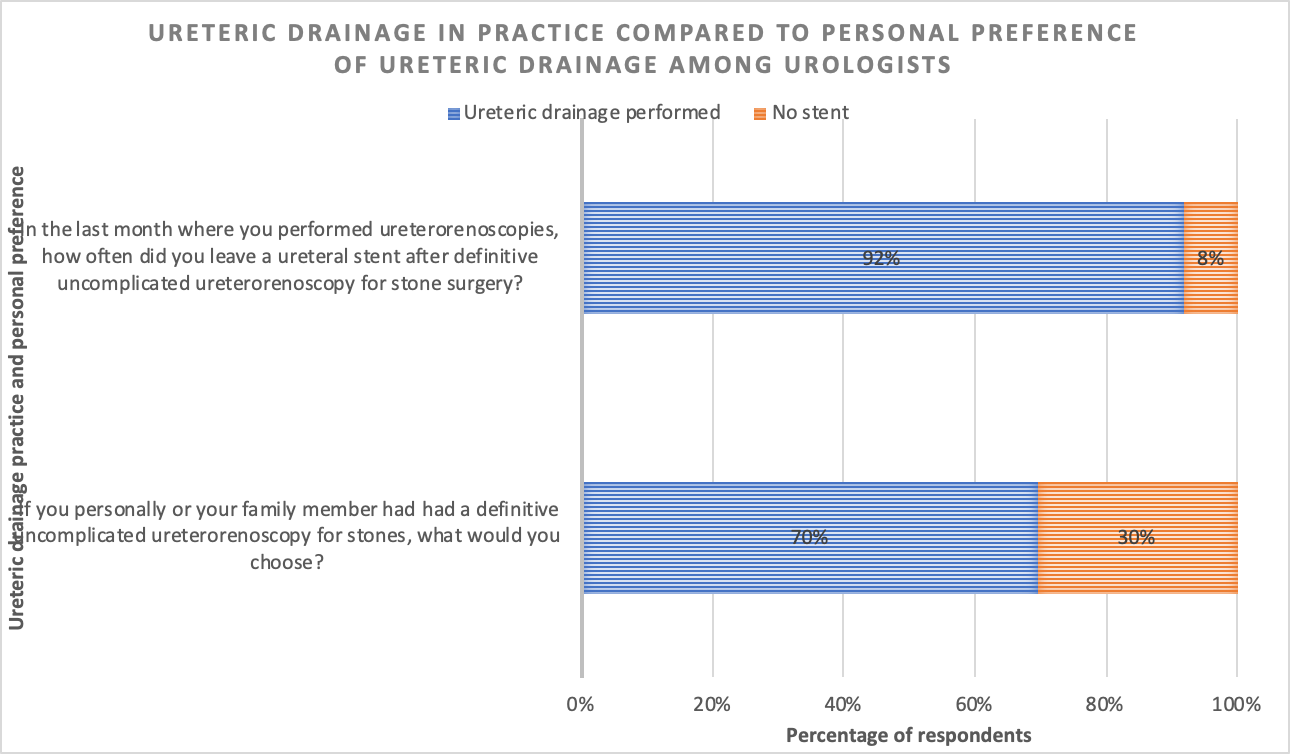


*Supplemental Figure C: Comparison of ureteric drainage practices after uncomplicated URS/FUR*
